# Spatial associations for slow musical tempos are revealed by increased stimulus density

**DOI:** 10.1038/s41598-026-49193-9

**Published:** 2026-04-28

**Authors:** Alberto Mariconda, Valter Prpic, Matteo De Tommaso, Mauro Murgia

**Affiliations:** 1 Department of Life Sciences, University of Trieste, Trieste, Italy; 2 Department of Theoretical and Applied Sciences, eCampus University, Novedrate, Italy; 3 Department of Psychology, University of Milan Bicocca, Milan, Italy

**Keywords:** Neuroscience, Psychology, Psychology

## Abstract

Recent evidence suggests the existence of spatial associations for musical tempo, characterized by faster left-key responses to relatively slow tempos and faster right-key responses to relatively fast tempos. However, while this effect has been consistently observed in the fast tempo range (133–201 bpm), previous studies investigating the slow tempo range (e.g., 40–104 bpm) have failed to find a reliable effect, reporting null or contradictory findings. The present study investigated whether a spatial association exists in the slow tempo range when specific methodological limitations (i.e. excessive gaps between stimuli) are addressed. Specifically, the study aimed to increase stimulus density through two complementary approaches: (1) testing the “absolute gap hypothesis” by using a physically linear scale with reduced intervals (“physical linearity condition”), and (2) testing the “proportional gap hypothesis” by using a perceptually linear scale based on Weber’s Law (“perceptual linearity condition”). The results revealed a significant spatial association in both conditions, demonstrating that a denser set of stimuli facilitates the emergence of spatial mappings regardless of the scaling method. These findings demonstrate that ensuring temporal continuity through increased stimulus density reveals a robust spatial association in the slow tempo range, thereby supporting a unified mechanism for temporal processing consistent with a generalized magnitude system.

## Introduction

An influential framework for understandings^1,2^. how the human mind processes different dimensions is A Theory of Magnitude (ATOM) model^3,4^. This model posits a shared, generalized magnitude system that jointly processes space, time, and quantity. A direct consequence of this shared system is the emergence of response biases and interference between these dimensions.

The most robust and well-known evidence supporting this model is the Spatial–Numerical Association of Response Codes (SNARC) effect^5^, which demonstrates that numbers are mentally represented along a spatial axis. Although alternative accounts have been proposed (see the polarity correspondence principle^6^, the working-memory-based accounts^7^, the dual-route model^8^, and cognitive control perspectives^9^ this spatial association has since been extended beyond symbolic numbers to non-symbolic numerosity^10–12^(but see also^13^ and a wide array of other non-numerical magnitudes. For instance, these effects, collectively known as SNARC-like effects, have been observed for physical magnitudes like luminance (where darker stimuli are associated with the left and brighter ones with the right)^14^, size (where smaller objects are associated to the left and larger ones to the right)^15,16^, and for facial expression of emotions (where more negative emotions are associated with the left and more positive emotions with the right)^17–20^. This body of work supports the ATOM model prediction that any type of quantity can be spatially coded (for an overview, see^21^.

The representation of time also falls within the ATOM framework. A growing body of research in cognitive psychology suggests that people mentally represent time along a spatial continuum, often described as a “Mental Timeline” (MTL) oriented from left to right^22^. This time–space association is deeply influenced by embodied and cultural factors, such as the dominant direction of writing and reading^23,24^. In the absence of specific manipulations, left-to-right readers associate the past (or short durations) with the left and the future (or long durations) with the right. However, time can also be represented along the sagittal axis (in front–behind), with cultural background significantly shaping whether the past or future is perceived as being in front of the observer (for an in front–behind spatial representation of time and the influence of the culture in the formation of this representation, see^25,26^.

This interplay between time and space is commonly observed via response time (RT) advantages in experimental tasks. For instance, studies using verbal stimuli have shown faster left-key responses for past-related words and faster right-key responses for future-related words^25,27,28^.

(for an overview see^29^. A similar pattern has been found for perceptual durations^30^. Such spatial associations have also been identified along vertical and diagonal axes^31,32^. Additionally, some evidence indicates a left-key advantage for “early” temporal stimuli and a right-key advantage for “late” temporal stimuli within a sequence^33,34^.

Particularly relevant to the present work are SNARC-like effects in the auditory context, demonstrating that various features of sound are spatially mapped. These include properties like pitch^35,36^, loudness^37^, and tempo^1,2,38^. The present study focuses specifically on tempo, defined as the speed of an auditory sequence, typically measured in beats per minute (bpm). De Tommaso and Prpic^1^ systematically investigated the SNARC-like effect for tempo by testing a full range (40–200 bpm; Experiment 1), a slow range (40–104 bpm; Experiment 2), and a fast range (133–201 bpm; Experiment 3), asking participants to evaluate whether a target sequence of beats was faster or slower than a reference sequence. Surprisingly, a clear effect emerged only in the fast tempo range. These findings suggest that, similar to other continuous quantities, tempo is spatially coded. Yet, unlike other spatial continua, tempo may operate within a restricted range of stimuli, implying that slow and fast tempos could be represented differently through space.

This unexpected result has generated a debate. Wood et al.^36^ raised methodological concerns on the work by De Tommaso and Prpic^1^, suggesting that participants in the slow-tempo conditions might have engaged in “anticipatory responding”, namely, made judgment before hearing the full target sequence. They argued that this could cause a shift in focus from the overall temporal speed (tempo) to the interval duration between beats, thereby masking the spatial effect. To address this, Mariconda et al.^2^, using a novel two-beat paradigm, confirmed that the effect is indeed driven by temporal speed, not the interval duration between beats. However, their study was limited to fast tempos, leaving the question of slow tempos unresolved.

Subsequently, Mariconda et al.^2^ revisited the study by De Tommaso and Prpic^1^, (1) to test the robustness of the SNARC-like effect for tempo and (2) to address the issues raised of previous studies, further investigating the question of slow tempos. To do this, they firstly modified the full-range condition (Experiment 1) by reducing the gap between stimuli (using 10 stimuli instead of 4 as in the original study). Indeed, they hypothesized that the large gaps in the original study had prevented the temporal continuity of the sequence, thereby leading to the absence of a SNARC-like effect. With this manipulation, they successfully found a spatial association in the full tempo range (40–200 bpm). However, their Experiment 2, which used a within-participants replication of the original study to test both the slow (40–104 bpm) and fast (133–201 bpm) tempo ranges, produced inconsistent findings for the slow tempo range. In the slow range (i.e. 40–104 bpm), the effect did not reach statistical significance, yet the overall analyses did not show significant differences between slow and fast tempi, indicating that a spatial association may still be plausible in the slow tempo range.

This absent (or weak) effect in the slow range is particularly surprising, as it seems to contradict a foundational principle of spatial–magnitude associations. SNARC and SNARC-like effects are typically understood to be driven by relative magnitude within a given context, not by absolute magnitude^5^(but see also^37^. For example, in the numerical domain, the number 4 is spatially associated with the right in a (0–5) range but with the left in a (4–9) range. Based on this “relativity principle”, the slow tempo range (40–104 bpm) should, in principle, have elicited a robust SNARC-like effect in previous studies, just as the fast range did^1,2^. The fact that it did not, or did so only weakly, suggests a boundary condition not previously observed.

To explain this weak (or absent) effect, Mariconda et al.^2^ hypothesized that the problem still lay with the large gaps between stimuli, consistently with the results of their Experiment 1, in which the reduction of the gaps between the stimuli was considered crucial for the occurrence of the SNARC-like effect in the full tempo range. Indeed, in percentage terms, a 16–bpm gap is much larger in the slow range (e.g., a 40% increase from 40 to 56 bpm) than in the fast range (e.g., less than 12% from 136 to 152 bpm). Consequently, these large proportional gaps might still be too large to create the necessary temporal continuity within the temporal sequence. Accordingly, the authors concluded that to strengthen and reliably detect the effect in the slow range, it would be necessary to increase the number of stimuli, consequently reducing the gaps and enhancing temporal continuity.

The present study directly follows the recommendation of Mariconda et al.^2^ to increase stimulus density and thereby enhance temporal continuity. The main aim was to test whether a reliable SNARC-like effect for tempo could emerge under these improved conditions. Specifically, we used eight target tempos (instead of four, as^1,2^) to create a denser and more continuous stimulus set, thereby testing the hypothesis that the weak (or absent) effect observed previously might have resulted from a methodological artifact caused by excessive gaps between temporal sequences.

Moreover, Mariconda et al.^2^ left unresolved a crucial methodological issue regarding whether temporal continuity depends on the absolute differences between tempos or on their relative, proportional differences. Crucially, these two possibilities do not represent competing hypotheses with diverging predictions; rather, they constitute two complementary methodological approaches. To address this, we tested the “Gap Hypothesis”, which posits that the emergence of a spatial–temporal association depends critically on stimulus density (i.e., reduced gaps between stimuli). Specifically, we tested two conditions replicating the slow tempo range previously investigated by De Tommaso and Prpic^1^ and Mariconda et al.^2^, to determine whether the critical factor for temporal continuity is the absolute difference or the proportional difference between tempos, as detailed in the following section.

### The present study

To disentangle the methodological issues raised in previous research, the present study involved two conditions tested within the same experimental session.

“The physical linearity condition” investigated the potential existence of a SNARC-like effect specifically within a slow tempo range, building on previous studies^1,2^. In those works, no effect^1^ or contradictory results^2^ emerged, probably due to the excessive gaps (i.e. 16 bpm) between the stimuli employed (e.g., 40 bpm, 56 bpm, 88 bpm, 104 bpm). Our hypothesis, consistent with previous results and based mainly on perceptual principles like Gestalt proximity rather than on the SNARC literature, was that these large gaps between stimuli hindered participants from forming a continuous mental representation of the sequence. To test this hypothesis, the gap between tempos was reduced by adopting a constant increment of 8 bpm between tempos. Specifically, this approach tests the “absolute gap hypothesis”: by halving the physical gaps used previously (from 16 bpm to 8 bpm), we created a denser, “physically linear” scale (i.e., 40, 48, 56, 64, 80, 88, 96, 104 bpm) to determine if it was sufficient to establish the temporal continuity required to elicit a SNARC-like effect.

In a complementary approach, the “perceptual linearity condition” was designed to investigate the role of perceptual linearity, contrasting with the physical linearity (absolute gap hypothesis) tested in the physical linearity condition. This study addresses a fundamental psychophysical aspect of time perception often overlooked in standard linear designs: the fact that a constant absolute difference (e.g., 8 bpm) is not perceived uniformly across the range. According to Weber’s Law, an 8–bpm increase constitutes a salient change at lower tempos (e.g., 40 bpm) but a negligible one at higher tempos (e.g., 100 bpm). Consequently, the perceptual linearity condition tested the “proportional gap hypothesis” by spacing tempos with a constant percentage increment. This manipulation yielded a “perceptually linear” scale, as opposed to a physically linear one, aiming to determine whether a sequence that respects the psychophysical nature of tempo perception provides the optimal conditions for eliciting a robust SNARC-like effect.

## Method

### Participants

A total of 61 university students (58 females, 3 males; *M*_*age*_ = 20.80 years, *SD* = 5.08) took part in the study. All participants were Italian nationals, born in Italy, and recruited from the psychology department (i.e. Department of Life Sciences) of the University of Trieste. Regarding handedness, 56 participants were right-handed and 5 were left-handed.

The sample size was determined a priori using G*Power software^41,42^. The power analysis (for a two-tailed, one-sample *t*-test on the mean of regression slopes) was set with a power of 80%, a significance level (α) of 0.05, and an expected effect size (Cohen’s d) of 0.395, based on a previous study^2^. Although the G*Power calculation indicated a minimum required sample size of 42 participants, we decided to recruit 61 students. This decision was motivated by the hypothesis that, in the study by Mariconda et al.^2^, the slow tempo range might elicit a small effect. Consequently, testing a larger sample increased the likelihood of detecting the SNARC-like effect.

The participants received academic credits for their participation. Inclusion criteria required a left-to-right reading/writing direction, normal hearing, and normal or corrected-to-normal vision. No participant reported being a musician or playing a musical instrument even at an amateur level (excluding any potential influence of formal or informal musical training on the results). Furthermore, all confirmed that their psychophysiological state was not affected by alcohol consumption or insufficient sleep in the preceding 24 hours^43^. The study received approval from the University of Trieste Ethics Committee (Prot. 145 1/2025) and was conducted in accordance with the ethical standards of the Declaration of Helsinki. Each participant provided written informed consent before beginning the experiment. Data collection took place in October 2025.

### Apparatus and stimuli

The experiment was programmed using Psychopy software version 3.0^44^ and run on a PC equipped with a 10th generation Intel Core i5 processor, 8 GB of RAM, and the Windows 10 operating system. Auditory stimuli were presented binaurally via noise-cancelling headphones (Sony MDR-ZX110NA), with the volume set to a comfortable level and kept constant for everyone. For response collection, a five-key response box was used; participants were specifically instructed to use only leftmost (first) and rightmost (fifth) keys.

The stimuli consisted of isochronous sequences of atonal metronome clicks, synthesized using Audacity software. These sounds were non melodic and atonal to avoid any musical or emotional confounding variables. In detail, nine audio files were used as stimuli for each condition, each with a duration of 3000 ms. These files were identical for all participants with only the inter-onset interval between clicks manipulated to vary the tempo. The 72–bpm sequence served as the reference stimulus in both conditions, while the remaining eight sequences were used as target stimuli. Regarding the specific characteristics of the conditions, in the physical linearity condition the tempos ranged from 40 to 104 bpm with a constant 8–bpm increment (i.e., 40, 48, 56, 64, 72, 80, 88, 96, and 104 bpm). Conversely, in the perceptual linearity condition, the apparatus and the general features of the stimuli (i.e., the 3000 ms duration and the acoustic properties of the clicks) remained unchanged from the physical linearity condition, but the crucial manipulation concerned the interval spacing. Unlike the physical linearity condition, where the fixed 8–bpm increment resulted in a decreasing percentage change across the scale (dropping from 20% between the slowest items to 8% between the fastest), the perceptual linearity condition employed a constant percentage increment of approximately 10.6% based on Weber’s Law. Consequently, while the relative difference remained stable, the absolute intervals progressively widened as the tempo increased. To ensure precision while maintaining ecologically valid tempo settings, the resulting values were rounded to the nearest integer. The final tempo sequences were: 48, 53, 59, 65, 72 (reference), 80, 88, 97, and 108 bpm. This spacing resulted in absolute differences ranging from 5 bpm at the lower end (48 to 53 bpm) to 11 bpm at the upper end (97 to 108 bpm). All auditory stimuli are available in the “OSF Storage” repository: https://osf.io/94xg3/overview? view_only=f02bc144d76a4bd68cc643023ef5d96c.

### Procedure

The experiment was carried out in a quiet environment, with participants tested one at a time to maintain controlled conditions. The experimental setup required participants to sit centrally in front of the screen, with the response box placed along their body’s midline. Participants were asked to minimize physical movement and position their index fingers on the two outer keys of the response box (the first key on the left and the fifth key on the right). The instructions stressed the importance of responding as quickly and accurately as possible.

Specifically, the experimental task required participants to judge whether the tempo of the target sequence was slower or faster than that of the reference sequence. Responses were made by pressing the designated key as quickly as possible. Participants completed both the physical linearity condition and the perceptual linearity condition in a single session. To avoid order effects, the presentation order of the two conditions was counterbalanced across the sample: half of the participants completed the physical linearity condition first, while the other half started with the perceptual linearity condition. Optional rest periods were provided between the two conditions and at the end of each experimental block to prevent fatigue.

Within each condition, the task consisted of two distinct blocks based on spatial–temporal compatibility: a congruent block and an incongruent block. In the congruent block, the left-key was assigned to the “slower” tempo and the right-key to the “faster” tempo. Conversely, in the incongruent block, the mapping was reversed (left-key for “faster”, right-key for “slower”). To avoid order effects, the presentation order of the blocks was counterbalanced across the sample (Version A: Congruent first; Version B: Incongruent first). Crucially, each block (Congruent and Incongruent) within each Condition consisted of two phases: a practice phase and an experimental phase. The block began with on-screen instructions regarding the key assignments, followed by a practice phase of 8 randomized trials (one for each target tempo) featuring immediate visual feedback to ensure the mapping was understood. Upon completion of the practice trials, participants proceeded immediately to the experimental phase, which consisted of 64 trials (8 repetitions for each of the 8 target tempos) presented in a randomized order. In total, for each Condition, participants completed a total of 144 trials (16 practice trials and 128 experimental trials across the two blocks).

The structure of each trial was identical in both the practice and experimental phases. A fixation cross appeared for 1500 ms, followed by the 3000 ms reference sequence (accompanied by the text “Reference” on screen). After a variable inter-stimulus interval (a fixation cross lasting randomly either 700 or 1000 ms), the target sequence was presented for 3000 ms together with the text “Target”. Participants were able to enter their response immediately upon the onset of the target stimulus.

Overall, the procedure largely replicated the procedure used by De Tommaso and Prpic^1^(Experiment 1) and Mariconda et al.^2^ (Experiment 2). However, specific parameters were modified for the current experiment: first, the number of tempos was increased from four to eight, resulting in a denser “physically linear” scale and thereby reducing the interstimulus interval from 16 to 8 bpm. Second, to enhance statistical power, a substantially larger sample (61) was recruited compared to those tested by De Tommaso and Prpic^1^(18) and Mariconda et al.^2^ (31).

The entire experimental session, including instructions, practice phases, lasted approximately 45 min.

### Data analysis

The experimental design involved the following within-subjects variables: Condition (two levels: physical linearity vs. perceptual linearity), Response keys (two levels: left vs. right), and Tempo (eight levels: from slowest to fastest). Specifically, for the physical linearity condition, Tempo levels were 40, 48, 56, 64, 80, 88, 96, and 104 bpm, whereas for the perceptual linearity condition, Tempo levels were 48, 53, 59, 65, 80, 88, 97, and 108 bpm. Additionally, Congruency (two levels: congruent vs. incongruent) was defined by the mapping between Response keys and Tempo, as described in the Procedure section. Specifically, data from the counterbalanced mapping blocks were coded by Response keys (left vs. right) to directly test the Tempo × Response keys interaction. Consequently, the statistical analysis considered were Response keys, Tempo, and Condition along with their interactions.

To account for the repeated-measures structure of the data and to address the redundancy of separate regressions, we employed Linear Mixed-Effects Models (LMMs) for all statistical analyses, using the lme4 and lmerTest packages in R. This approach allowed for a direct estimation of fixed effects while treating participants as a random factor. Model selection was performed by comparing models of increasing complexity using the Akaike Information Criterion (AIC) and Likelihood Ratio Tests.

First, we analysed the two experimental conditions separately. For each condition, the model included Response keys and Tempo as fixed factors, along with their interaction (Tempo × Response keys). We evaluated the results by first assessing the overall significance of the main effects and the interaction via the Type III Analysis of Variance table (based on *F*-tests using Satterthwaite’s method). Significant interactions were then interpreted by inspecting the table of Fixed Effects coefficients to determine the direction of the spatial–temporal association. In this model, the reference baseline was explicitly defined as the left-key response at the slowest Tempo level (Tempo 1). Therefore, the interaction coefficients quantify how the difference between response keys changes at faster tempos relative to this specific starting point. Specifically, a negative interaction coefficient for the fastest tempos (associated with a significant *t-*test) would indicate that the right-key becomes significantly faster than the left-key as tempo increases, effectively reversing the pattern observed at the slowest tempo and confirming the SNARC-like effect.

Subsequently, to directly compare the two conditions, we fitted a unified LMM on the entire dataset. Since the specific BPM values differed between conditions (i.e., 40–104 bpm for physical vs. 48–108 bpm for perceptual), the tempo levels were recoded into ordinal steps from Tempo 1 (slowest) to Tempo 8 (fastest) to allow for a direct comparison within the same model. This unified model included Response keys, Tempo (levels 1–8) and Condition as fixed factors, along with all their interactions. The three-way interaction (Condition × Response keys × Tempo) was specifically tested to assess whether the spatial–temporal association differed between the physical and perceptual linearity conditions.

Prior to conducting data analysis, the data were screened to remove incorrect responses and outliers following the criteria used by Mariconda et al.^2^. Accordingly, outliers were defined as RTs faster than 120 ms or exceeding the participant’s mean by more than three standard deviations. Notably, no participants were excluded from the final sample, as none exceeded the exclusion threshold for error rates (> 20%) or exhibited over 50% of missing values within any specific condition. These criteria ensured that the RTs reflected stable performance rather than random guessing near the chance level (50%), which would introduce noise and compromise the validity of the response time data. Overall, in the physical linearity condition incorrect responses were 4.12% and outliers 1.67%; in the perceptual linearity condition incorrect responses were 4.62% and outliers 1.54%.

## Results

**Fig. 1:**
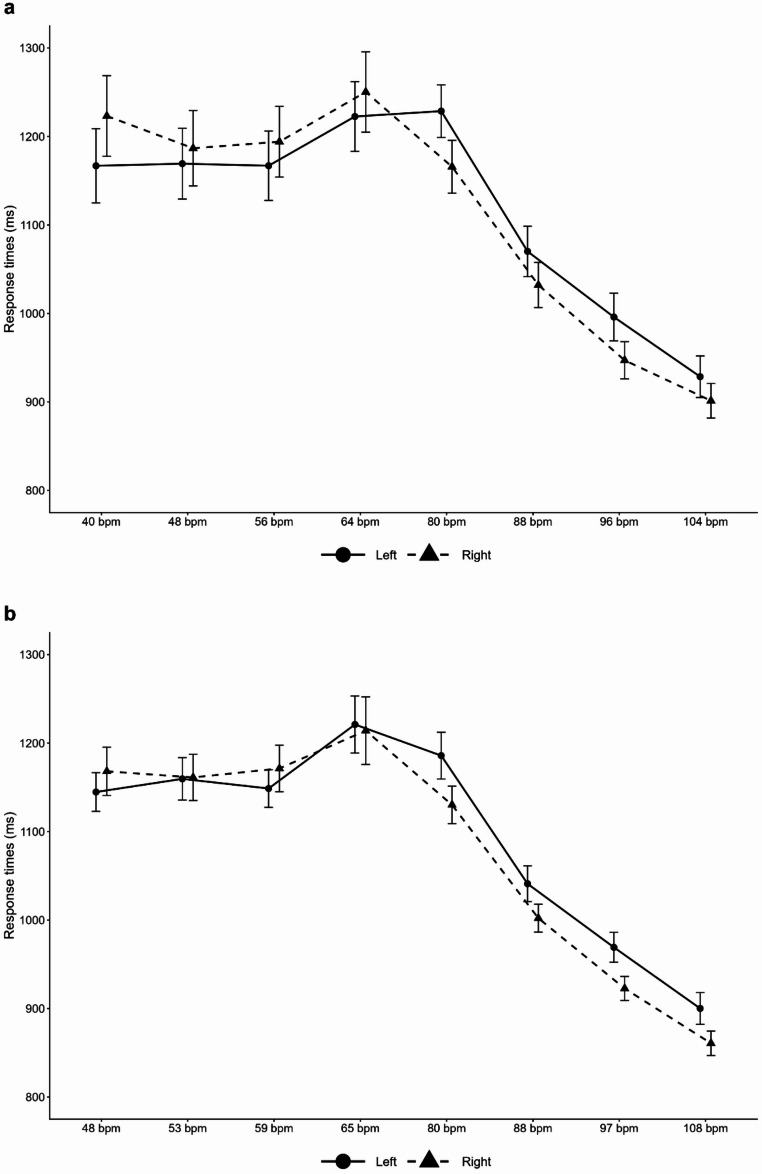
Results of the Linear Mixed-Effects Models analysis for the *physical linearity condition* (Panel **A**) and the *perceptual linearity condition* (Panel **B**). The line plots display the Estimated Marginal Means (EMMeans) of Response Times (RTs) as a function of Tempo and Response key. Error bars represent standard errors derived from the model. In both conditions, the interaction pattern illustrates a transition from a left-key advantage at slower tempos to a right-key advantage at faster tempos.

The analysis first focused on the physical linearity condition. Model selection based on AIC indicated that the maximal model, including by-participant random intercepts and random slopes for Response keys, Tempo, and their interaction, provided the best fit to the data. This model showed a good goodness-of-fit (*R*²*m* = 0.14, *R*²*c* = 0.62). The analysis revealed a significant main effect of Tempo (*F*(7, 426.08) = 90.84, *p* <.001), while the main effect of Response keys was not significant (*F*(1, 476.48) = 0.87, *p* =.350). Specifically, the inspection of fixed effects for the main effect of Tempo confirmed that participants responded significantly faster to the fastest stimuli (i.e., tempos > 72 bpm) compared to the slowest baseline (40 bpm; e.g., at 104 bpm: *b* = − 0.238, *p* <.001). This pattern likely reflects the temporal structure of the stimuli: acoustic information required for decision-making becomes available earlier in faster sequences compared to slower ones, which necessitate a longer listening duration. Crucially, a significant Tempo × Response keys interaction emerged (*F*(7, 475.68) = 5.61, *p* <.001), highlighting a specific spatial-temporal mapping (Fig. 1a). Specifically, the analysis of fixed effects showed a clear transition: at the slowest baseline (Tempo 1), responses were significantly slower with the right-key compared to the left (*b* = 0.056, *p* =.002), indicating a left-key advantage. As tempo increased, the Tempo × Response keys interaction coefficients became progressively negative, indicating a shift in performance. This shift culminated in the fastest tempos (e.g., Tempo 5 to 8), where the right-key became significantly faster than the left, fully reversing the pattern observed at the baseline. These results support the “absolute gap hypothesis”, revealing a significant SNARC-like effect (see Fig. 1a). This finding suggests that the null or contradictory results reported in previous studies were likely attributable to excessive interstimulus intervals (16 bpm), which hindered the formation of a continuous mental representation. In line with the Gestalt principle of proximity, the reduction of these gaps appears to have established the temporal continuity necessary to elicit spatial associations. Specifically, the observation that participants responded faster with the left-key to slower tempos and with the right-key to faster tempos demonstrates that reduced spacing successfully facilitates a spatial–temporal mapping.

Subsequently, the analysis examined the perceptual linearity condition. Similarly, the maximal model structure provided the best fit to the data, explaining a substantial proportion of variance (*R*²*m* = 0.13, *R*²*c* = 0.48). The analysis revealed a significant main effect for Tempo (*F*(7, 412.84) = 145.42, *p* <.001) and Response keys (*F*(1, 470.53) = 7.09, *p* =.008). Furthermore, a significant interaction between Tempo and Response keys emerged (*F*(7, 469.42) = 2.91, *p* =.006). Specifically, the inspection of fixed effects for the main effect of Tempo confirmed that participants responded significantly faster to the fastest stimuli (i.e., tempos > 72 bpm; Fig. 1b) compared to the slowest baseline (48 bpm; e.g., at 108 bpm: *b* = − 0.245, *p* <.001). As in Experiment 1, this pattern is likely attributable to the temporal structure of the acoustic stimuli, where decision-relevant information becomes available earlier in faster sequences. Additionally, the main effect of Response keys indicated a general advantage for right-key responses. Most importantly, the interaction confirmed the spatial nature of the effect. Specifically, the analysis of fixed effects showed a clear transition: while at the slowest baseline (Tempo 1) no significant asymmetry was observed (*b* = 0.023, *p* =.203), the Tempo × Response keys interaction coefficients became progressively negative as tempo increased. This shift culminated in the fastest tempos (e.g., Tempo 5 to 8), where the right-key became significantly faster than the left (e.g., Tempo 5: *b* = − 0.079, *p* =.003), replicating the pattern observed in the physical linearity condition. This pattern confirms the presence of a SNARC-like effect even when using a perceptually linear scale (see Fig. 1b).

### Comparison between conditions

Given that a significant SNARC-like effect was observed in both experimental conditions, we performed additional analyses to assess the intraindividual consistency and relative strength of the effect. First, to test the intraindividual consistency, we performed a correlation analysis between the regression slopes (*b* coefficients) obtained in the physical linearity condition and in the perceptual linearity condition. A significant positive correlation was found (*r* =.56, *p* <.001), indicating that participants who exhibited a stronger SNARC-like effect in the physically linear condition also tended to show a stronger effect in the perceptually linear condition. This relationship remained significant even when using a non-parametric test robust to outliers (*r*_s_ = 0.29, *p* =.025).

Beyond this intraindividual consistency, we explicitly tested whether the overall magnitude of the spatial association differed between the two conditions by inspecting the unified Linear Mixed-Effects Model fitted on the entire dataset (see Data Analysis). The unified model showed a good fit to the data (*R*²*m* = 0.14, *R*²*c* = 0.62). Crucially for this purpose, the three-way interaction (Condition × Response keys × Tempo) was not significant (*F*(7, 1301.04) = 0.42, *p* =.888), indicating that the strength and nature of the SNARC-like effect did not statistically differ between the physical and perceptual linearity conditions. Taken together, these findings demonstrate that (1) individual sensitivity to the effect is stable across tasks and (2) the specific type of linearity (physical vs. perceptual) does not significantly modulate the strength of the spatial association, suggesting that stimulus density is the key factor for ensuring temporal continuity and eliciting the SNARC-like effect.

### General discussion

The main aim of the present study was to investigate whether the SNARC-like effect for tempo extends to the slow tempo range (40–104 bpm), a question that has remained controversial in the literature. While the spatial mapping of fast tempos is well-established, previous research failed to find a reliable effect for slow tempos^1^ or reported contradictory findings^2^. Following the “Gap Hypothesis” proposed by Mariconda et al.^2^, we hypothesized that these null results were not due to a lack of spatial representation for slow tempos, but rather to a methodological limitation: the excessive distance between stimuli (i.e., large gaps) prevented the formation of the temporal continuity necessary to represent the sequence as a continuous spatial magnitude. Accordingly, we proposed that high stimulus density should induce the temporal continuity. For instance, we operationally define this continuity as the functional link between stimulus density (a physical property) and the emergence of the SNARC-like effect (a behavioral observation), suggesting that the spatial–temporal association requires a continuous magnitude representation rather than isolated temporal units.”

To test this, we increased the stimulus density (using 8 tempos instead of 4), thereby reducing the gaps, and tested two types of linearity: physical linearity (i.e. the physical linearity condition, with constant absolute increments) and perceptual linearity (i.e. the perceptual linearity condition, with constant proportional increments). The results confirmed our hypothesis: a significant SNARC-like effect emerged in the slow tempo range in both conditions. Participants responded faster with the left-key to slower tempos and with the right-key to faster tempos. This finding provides the first robust evidence that slow music tempos are spatially represented, resolving the inconsistencies of previous studies.

The emergence of the effect in both conditions suggests that the critical factors for eliciting a SNARC-like effect in the slow range were the increased stimulus density and the enhanced statistical power. Crucially, the data demonstrate that both the constant absolute gap (8 bpm in the physical linearity condition) and the constant proportional gap (~ 10.6% in the perceptual linearity condition) successfully reduced the intervals between tempos, allowing the formation of a continuous mental timeline, overcoming the fragmentation likely caused by the large gaps in previous studies^1,2^. Finally, sample size likely played a key role. Previous studies failing to detect this effect (e.g^1^.) relied on small samples (*N* = 18). With 61 participants, our study had sufficient statistical power to reveal an effect that was likely missed in previous work due to Type II errors.

Crucially, our analysis revealed that the strength of the spatial association did not significantly differ between the two conditions (as indicated by the non-significant three-way interaction in the unified LMM). This demonstrates that the spatial mapping of tempo is robust and persists regardless of whether the scale follows a simple arithmetic regularity (as in the physical linearity condition) or respects the non-linear nature of human perception (as in the perceptual linearity condition). Overall, these findings validate the Gap Hypothesis and the importance of adequate statistical power: when the auditory system is presented with a sufficiently dense sequence and tested with a robust sample, it successfully maps temporal magnitude onto a spatial continuum, regardless of whether the progression is arithmetic or geometric. This implies that the spatial mapping of tempo is primarily driven by ordinality (i.e., relative speed) rather than by the specific metric properties of the intervals.

Finally, regarding the main effect of Response keys, a general right-key advantage emerged in the perceptual linearity condition but not in the physical linearity condition. We interpret this asymmetry as resulting from the specific interaction patterns observed at the slowest tempos. In the physical condition (range 40–104 bpm), the robust left-key advantage at the baseline effectively counterbalanced the right-key advantage at fast tempos, resulting in a null main effect. Conversely, in the perceptual condition (range 48–108 bpm), the absence of a significant left-key advantage at the baseline meant that the right-key advantage at faster tempos was not offset, leading to a significant overall main effect likely driven by the participants’ right-handedness.

Theoretically, these results provide crucial support for the ATOM model^3,4^. The core premise of ATOM is the existence of a generalized magnitude system that processes space, time, and quantity via a common metric. Previous failures to detect spatial associations in the slow tempo range posed a challenge to this framework, potentially implying a fractured system where different temporal ranges rely on distinct processing mechanisms. By demonstrating that spatial associations exist for the full spectrum of musical tempo, including the previously elusive slow range, our findings address this theoretical issue. Indeed, we provide evidence that the spatial representation of tempo is unified, operating consistently across both slow and fast speeds. Overall, our findings support that temporal magnitude is processed by a single, general mechanism, as predicted by the ATOM model and consistent with findings for other non-numerical magnitudes like size and pitch^16,35,36,45^.

Notably, while our findings support the generalized magnitude system proposed by ATOM, the specific pattern of results suggests the involvement of additional coding mechanisms. First, the observed asymmetry–where the effect is particularly robust for relatively faster tempos (> 72 bpm)–aligns with the Polarity Correspondence Principle^6^. This framework posits that conceptual dimensions are coded as binary opposites consisting of an “unmarked” pole (coded as positive; e.g., “Fast”, “Right”) and a “marked” pole (coded as negative; e.g., “Slow”, “Left”). Consequently, the mapping is inherently more robust when two positive codes coincide (i.e., Fast + Right), as they enjoy a processing advantage compared to the alignment of two negative codes (i.e., Slow + Left). Thus, while increasing stimulus density enabled the emergence of the mapping in the slow tempo range (i.e. 40–108 bpm), this structural asymmetry in polarity coding likely contributes to the superior strength of the effect observed for the relatively faster tempos (i.e. 72–108 bpm) compared to the relatively slower ones (i.e. 40–72 bpm).

Second, regarding the shape of the spatial–temporal association (Fig. 1), our results display a step-like profile rather than a continuous linear trend. This pattern is consistent with the computational model of the SNARC effect^8^, which suggests that the shape of the spatial association depends on the task: while implicit tasks (e.g., parity judgments) tend to elicit continuous linear associations, explicit magnitude classification tasks (such as our “slower vs. faster” discrimination) activate categorical spatial coding. Thus, the observed “all-or-none” trend reflects the categorical nature of the decision process, consistent with the specific demands of an explicit classification task.

A potential limitation of the present study concerns the relationship between tempo and numerosity. Since the duration of the stimuli was kept constant (3000 ms) to avoid confounding effects related to duration itself, the number of beats necessarily varied across stimuli (i.e., fewer beats for slower tempos, more beats for faster tempos). Consequently, one could argue that the observed effect might be driven by numerosity (a standard SNARC effect) rather than tempo. However, we consider this interpretation unlikely for two main reasons. First, in the slow tempo range investigated here (40–104 bpm), the absolute variation in the number of beats is relatively small (ranging approximately from 2 to 5 beats). It is debatable whether such a small numerical range would be sufficient to drive the robust spatial effects observed, typically associated with larger numerosity differences. Second, and perhaps more importantly, in a naturalistic musical context, tempo and numerosity are intrinsically linked: within a fixed time window, a faster tempo is a sequence with more events. Attempting to dissociate them completely often results in artificial stimuli that lack ecological validity. Furthermore, previous research using a fixed-numerosity paradigm^38^ demonstrated that the SNARC-like effect for tempo persists even when numerosity is controlled. This strongly suggests that while numerosity and tempo were correlated in our task, tempo itself acts as a distinct magnitude capable of eliciting independent spatial associations.

Another limitation pertains to the difficulty of disentangling the specific contributions of stimulus density and statistical power. Since the increase in stimulus density (from 4 to 8 tempos) was implemented simultaneously with an increase in sample size relative to previous work^1,2^, we cannot definitively rule out that the observed results reflect a combination of restored temporal continuity and increased statistical power, rather than density alone. Future research could address this by orthogonally manipulating density and sample size to isolate their respective roles.

Finally, a further limitation concerns the overlap between the stimulus sets. Although the physical and perceptual conditions were designed to test distinct hypotheses (absolute vs. proportional gaps), the constraint of using the same reference tempo (72 bpm) and a comparable overall range resulted in a partial overlap of specific values (i.e., 48, 72, 80, and 88 bpm were identical in both sets). This structural similarity might have reduced the empirical contrast between the two conditions, potentially contributing to the similar interaction patterns observed. Future research aiming to dissociate the absolute and proportional gap hypotheses more sharply should implement tempo distributions that diverge more strongly while preserving the intended theoretical distinction.

Overall, considering these findings, future research could further investigate whether musical expertise enhances the strength of the spatial representation of tempo. Since this relationship has not yet been explored, it remains to be seen whether the extensive training of musicians results in a more robust or automated spatial coding, particularly within the perceptually complex slow range.

### Analysis of order effects

Since recent evidence showed that task order and sequence-related factors can modulate spatial–magnitude associations^46^, we conducted supplementary analyses using Linear Mixed-Effects Models (LMMs) to explicitly test whether the order of presentation influenced our main results.

First, regarding the Task Order (i.e., whether participants performed the Physical Linearity condition or the Perceptual Linearity condition first), we fitted a unified model (with data from both condition) including Task Order as a between-subjects factor. The analysis revealed that the critical three-way interaction (Condition × Response keys × Task Order) was clearly non-significant (F(7, 466.36) = 0.45, *p* =.873), confirming that the presentation order of the two conditions did not modulate the spatial–temporal association.

Second, regarding the Block Order (i.e., whether participants performed the Congruent or Incongruent block first within each condition), we tested its potential influence by adding Block Order as a fixed factor. The analysis showed that the three-way interaction (Tempo × Response keys × Block Order) was not significant (F(7, 467.85) = 1.95, *p* =.060), indicating that the strength of the SNARC-like effect was not significantly dependent on whether participants started with a congruent or incongruent mapping.

Overall, these control analyses confirm that the reported SNARC-like effects are robust and not driven by carry-over effects related to the experimental order.

## Conclusion

In conclusion, the present study resolves the debate regarding the spatial representation of slow tempos. We demonstrated that when stimuli are presented with sufficient density to ensure temporal continuity , a reliable SNARC-like effect emerges for slow tempos, just as it does for fast ones. This effect is robust across both physically and perceptually linear scales. These findings not only support the ATOM model but also highlight the importance of psychophysical parameters, specifically stimulus density, in the experimental design of (temporal) magnitude mappings.

## Data Availability

To ensure full transparency and reproducibility, the complete statistical outputs for all analyses reported in this study—including the main models and the control analyses for Task Order and Block Order reported in the Supplementary Materials—along with the raw data and stimuli, are available in OSF (Open Science Framework) repository: [https://osf.io/94xg3/overview? view\_only=f02bc144d76a4bd68cc643023ef5d96c](https:/osf.io/94xg3/overview? view_only=f02bc144d76a4bd68cc643023ef5d96c).
